# Different Distribution of HIV-1 Subtype and Drug Resistance Were Found among Treatment Naïve Individuals in Henan, Guangxi, and Yunnan Province of China

**DOI:** 10.1371/journal.pone.0075777

**Published:** 2013-10-03

**Authors:** Lin Li, Guoqing Sun, Shujia Liang, Jianjian Li, Tianyi Li, Zhe Wang, Wei Liu, Shaomin Yang, Yongjian Liu, Xiaolin Wang, Jingyun Li

**Affiliations:** 1 Department of AIDS Research, State Key Laboratory of Pathogen and Biosecurity, Beijing Institute of Microbiology and Epidemiology, Beijing, China; 2 Henan Center for Disease Control and Prevention, Zhengzhou, Henan, China; 3 Guangxi Center for Disease Prevention and Control, Nanning, China; 4 Yunnan Provincial Hospital Infectious Disease, AIDS Care Center (YNACC), Kunming, Yunnan, China; Chinese Academy of Sciences, Wuhan Institute of Virology, China

## Abstract

Yunnan, Guangxi and Henan are the provinces with the most severe HIV epidemic in China, which were also among the first group of areas providing free ART in 2004. However, little comprehensive data are available on prevalence of HIV subtype and baseline drug resistance in drug-naïve populations. In this study, 1746 treatment-naïve HIV-positive individuals were randomly selected from new-reported cases in Henan, Guangxi and Yunnan. Among of them, subtypes and drug resistance of 1159 strains were determined by amplifying and sequencing full-length *pol* genes. Significantly different distributions of HIV subtypes prevalent in three provinces were identified (P<0.01). CRF08_BC was found dominant in Yunnan (59.8%), while CRF01_AE was dominant in Guangxi (77.3%) and subtype B was dominant in Henan province (93.9%). The total prevalence of drug resistance was 7.1%. The highest prevalence of HIV drug resistance was found in Henan (12.2%), followed by Yunnan (5.6%) and Guangxi (3.3%). The results of this study suggest that genetic drug-resistance should be tested before initiation of ART in China, especially in Henan province. Furthermore, the prevalence of HIV drug resistant strains should be considered separately in different areas in China before the change of different free ART regimens.

## Introduction

Highly active antiretroviral therapy (HAART) has resulted in marked improvements in morbidity and mortality from HIV-1 infection [1-3]. Nevertheless, it is still not curative. With the long-term use of HAART, HIV drug resistance occurs in a substantial proportion of treated patients and accumulates over time. Undoubtly, drug resistance has become one of the most profound limitations of current antiretroviral therapy, especially in developing countries with limited therapy resources [4]. In China, the ‘‘four free one care” policy was put forward in 2003 [5], free ART is one of “four free” which focused on improving the life of HIV/AIDS patients. The nationwide free ART reduced HIV/AIDS-related morbidity and mortality significantly. However, it is hard to fulfill surveillance on standard regimens due to the use of antiviral therapy in large scale and in short time, drug resistance is emerging and becoming severe in some areas [6].

Yunnan, Guangxi and Henan provinces are the provinces in China with the most severe HIV epidemic. Different epidemic patters were reported in those three provinces. Yunnan was considered as the epicenter of China characterized with most complicated HIV subtype distribution and prevalence of unique recombinant forms [7]. Guangxi was the province with most rapid increase of newly reported HIV-positive cases in recent years [8]. Henan province was characterized with HIV infection outbreak in the mid 1990s in former paid blood donors. Due to the severe conditions of HIV epidemic, those three provinces were among the first group of free ART provinces in 2004. All of registered patients with CD4+ T cells less than 200 cells/µl started to take drugs. Due to the difficulty in surveillance and short supply of medical resources in many areas, the prevalence of HIV drug resistance has been studied. According to a cohort study fulfilled in Henan rural area, the cumulative drug resistance of HIV reached to 88% after six years of HAART [6]. The latest studies fulfilled in some cities in Henan province revealed that HIV-1 drug resistance could be detected in 47.1% (178/378) patients with HAART [9]. With the cumulation of individuals containing drug-resistant HIV variants, transmitted drug-resistant (TDR) variants would be common.

Undoubtly, baseline resistance may reduce treatment efficacy, thus requiring the first line regimen to be adjusted accordingly. Up to now, free antiretroviral treatment (ART) has been available in these three provinces for 9 years. Although many studies have been fulfilled on prevalence of drug-resistant HIV variant, most of those studies were confined to some specific area or populations [10-13]. No comprehensive data are available on HIV subtypes or baseline prevalence of drug resistance in drug-naïve populations. With the scale-up of a free ART program, we conducted province wide surveys in Yunnan, Guangxi and Henan provinces to investigate the distribution of subtypes and prevalence of antiretroviral drug resistance of HIV strains in treatment-naive HIV-infected individuals.

## Materials and Methods

### Ethics Statement

The Ethical Review Board, Science and Technology Supervisory Committee at the Beijing Institute of Microbiology and Epidemiology approved this study. All participants provided their written informed consent to participate in the study.

### Study subjects and specimens

In Henan, Guangxi and Yunnan provinces, CD4+ T cell counts of all HIV-positive drug-naïve patients are required to be measured at least once each year. HIV-positive drug-naïve individuals which are 2-8% of total patients who went to the local center for disease prevention and control (CDC) for HIV diagnosis and CD4+ T cell count were recruited with written informed consents. Henan CDC, Guangxi CDC, and Yunnan AIDS care center were responsible for the training of staffs from the local CDC or AIDS care center. The staffs in local CDC who knew the objectives of the project and the strategy for enrollment were responsible for the investigation of demographical background and collection of peripheral blood. All blood samples were sent to Henan CDC, Guangxi CDC, and Yunnan AIDS care center within 24 hours of collection. CD4+ T-cells were calculated using flow cytometry with fresh whole blood samples in Henan CDC, Guangxi CDC, and Yunnan AIDS care center before plasma was separated and stored in -80°C freezer. After that, plasma samples were sent to Beijing Institute of Microbiology and Epidemiology to obtain RNA for further analysis.

### HIV-1 RNA extraction, amplification and sequencing

Viral RNA was extracted from 500µl HIV-1 positive plasma specimens with high pure viral RNA kit (Roche, USA). Viral full length *pol* genes were amplified separately by using reverse transcriptional nested PCR as described before [14]. Positive PCR products were sequenced by Huada genomics company (China) with a variety of internal specific primers (available on request) after being purified.

### Edit, assemble of pol sequences and HIV-1 drug resistance analysis

All of the sequenced fragments were edited and assembled as described before [15]. To check for potential contamination, the sequences obtained were compared to all known sequences in the HIV database by a Basic Local Alignment Search Tool (BLAST) search (http://hiv-web.lanl.gov/content/index). Drug-resistance mutations (DRMs) profile and antiretroviral susceptibility were inferred using the World Health Organization 2009 list of mutations for surveillance of TDR as implemented in the Calibrated Population Resistance tool (v5.0 beta) [16,17] (http://hivdb.stanford.edu).

### HIV genotyping and phylogenetic analysis

HIV-1 genotype was determined using the national center for biotechnology information viral genotyping tool (http://www.ncbi.nih.gov/projects/genotyping/formpage.cgi), REGA (http://www.bioafrica.net/rega-enotype/html/subtypinghiv.html), and further confirmed by phylogenetic analysis with reference sequences using the Neighbor-Joining method in MEGA5.0 software [18]. The possible intertype mosaicism was screened with online Recombination Identification Program (RIP, version 3.0; http://hiv-web.lanl.gov) and further confirmed by the online software jpHMM-HIV (http://jphmm.gobics.de/).

### Statistical analysis

The 95% confidence interval of the prevalence rates of baseline drug resistance was calculated using the binomial distribution and the modified Wald method. Categorical variables were compared using the Fisher’s exact test or Chi-square test performed using SPSS (SPSS version 17.0, SPSS Inc).

## Results

### Characteristics of participants

A total of 1746 treatment-naïve HIV infected participants (including 324 individuals residing in Yunnan province, 676 individuals in Guangxi province and 746 individuals in Henan province) were enrolled into the study in 2009-2010. 1159 (66.4%) full length *pol* genes (including 251 sequences from Yunnan, 481 sequences from Guangxi, and 427 sequences from Henan) were successfully obtained with proper open reading frames. The inability to obtain the full length *pol* genes from the other samples may be due to low viral load, degradation of viral RNA during sample transportation or sequence variations at the primer binding sites. More sequences were obtained from individuals with low CD4+ cell counts. Basic Local Alignment Search Tool (BLAST) search showed no evidence of sample contamination. The baseline characteristics of those participants with full length *pol* gene were summarized in Table 1. The ethnic composition of participants was different among three provinces. Yunnan was characterized with comprehensive ethnics, while Guangxi was characterized with Zhuang ethnic and Henan with Han ethnic. The transmitting routes in three provinces were also different (Pearson Chi-square test, P<0.01). Multiple transmitting routes with heterosexual transmission as the dominant route were found in Yunnan and Guangxi, while blood-born route was found dominant in Henan. The median age of the participants was 44.7 years (ranging from 2 to 80 years). More participants were enrolled from male than female (61.2% versus 38.8%). The CD4 T cell counts varied widely from 4 to 1018 cells/µl.

**Table 1:** Characteristics of treatment-naïve HIV-1 infected participants with pol sequences.

Characteristics	Areas			Total
	Yunnan	Guangxi	Henan	
Participants	251 (100)*[21.7]**	481 (100) [41.5]	427 (100) [36.8]	1159 (100) [100]
Age [median (min–max)]	43.5 (3-80)	38.1 (3-76)	45.0 (3-78)	41.6 (3-80)
Sex				
Male	141 (56.2) [19.9]	328 (68.2) [46.3]	240 (56.2) [33.9]	709 (61.2) [100]
Female	110 (43.8) [24.4]	153 (31.8) [34.0]	187 (43.8) [41.6]	450 (38.8) [100]
Ethnic				
Han ethnic	176 (70.1) [23.0]	164 (34.1) [21.5]	424 (99.3) [55.5]	764 (65.9) [100]
Zhuang minority ethnic	0 (0.0) [0.0]	248 (51.6) [100]	0 (0.0) [0.0]	248 (21.4) [100]
Bai minority ethnic	8 (3.2) [100]	0 (0.0) [0.0]	0 (0.0) [0.0]	8 (0.7) [100]
Dai minority ethnic	11 (4.4) [91.7]	1 (0.2) [8.3]	0 (0.0) [0.0]	12 (1.0) [100]
Hani minority ethnic	6 (2.4) [100]	0 (0.0) [0.0]	0 (0.0) [0.0]	6 (0.5) [100]
Hui minority ethnic	7 (2.8) [77.8]	0 (0.0) [0.0]	2 (0.5) [22.2]	9 (0.8) [100]
Wa minority ethnic	24 (9.6) [100]	0 (0.0) [0.0]	0 (0.0) [0.0]	24 (2.1) [100]
Yi minority ethnic	6 (2.4) [100]	0 (0.0) [0.0]	0 (0.0) [0.0]	6 (0.5) [100]
Yao minority ethnic	0 (0.0) [0.0]	8 (1.7) [100]	0 (0.0) [0.0]	8 (0.7) [100]
Other minority ethnic	7 (2.8) [46.7]	7 (1.5) [46.7]	1 (0.2) [6.7]	15 (1.3) [100]
Unknown	6 (2.4) [10.2]	53 (11.0) [89.8]	0 (0.0) [0.0]	59 (5.1) [100]
Route of transmission				
Heterosexual (HST)	163 (64.9) [26.8]	374 (77.8) [61.4]	72 (16.9) [11.8]	609 (52.5) [100]
Homosexual (MSM)	2 (0.8) [7.1]	10 (2.1) [35.7]	16 (3.7) [57.1]	28 (2.4) [100]
Injection Drug user (IDU)	45 (17.9) [58.4]	32 (6.7) [41.6]	0 (0.0) [0.0]	77 (6.6) [100]
Blood-born (BB)	2 (0.8) [0.6]	0 (0.0) [0.0]	310 (72.6) [99.4]	312 (26.9) [100]
Mother to Children (MTC)	8 (3.2) [25.8]	6 (1.2) [19.4]	17 (4.0) [54.8]	31 (2.7) [100]
Unknown	31 (12.4) [30.4]	59 (12.3) [57.8]	12 (2.8) [11.8]	102 (8.8) [100]
CD4+ count(cells/µl)				
≤200	146 (58.2) [29.3]	205 (42.6) [41.1]	148 (34.7) [29.7]	499 (43.1) [100]
201-350	80 (31.9) [25.6]	116 (24.1) [37.1]	117 (27.4) [37.4]	313 (27.0) [100]
351-500	15 (6.0) [7.2]	110 (22.9) [52.9]	83 (19.4) [52.9]	208 (17.9) [100]
≥501	10 (4.0) [7.2]	50 (10.4) [36.0]	79 (18.5) [36.0]	139 (12.0) [100]

*Numbers in parentheses indicate the proportion of the HIV-infected in each subgroup as a percentage of the total for the area.

** Numbers in square brackets indicate the proportion of the HIV-infected in each subgroup as a percentage of the total for the characteristic group.

### Prevalence of HIV subtypes

**Figure 1:**
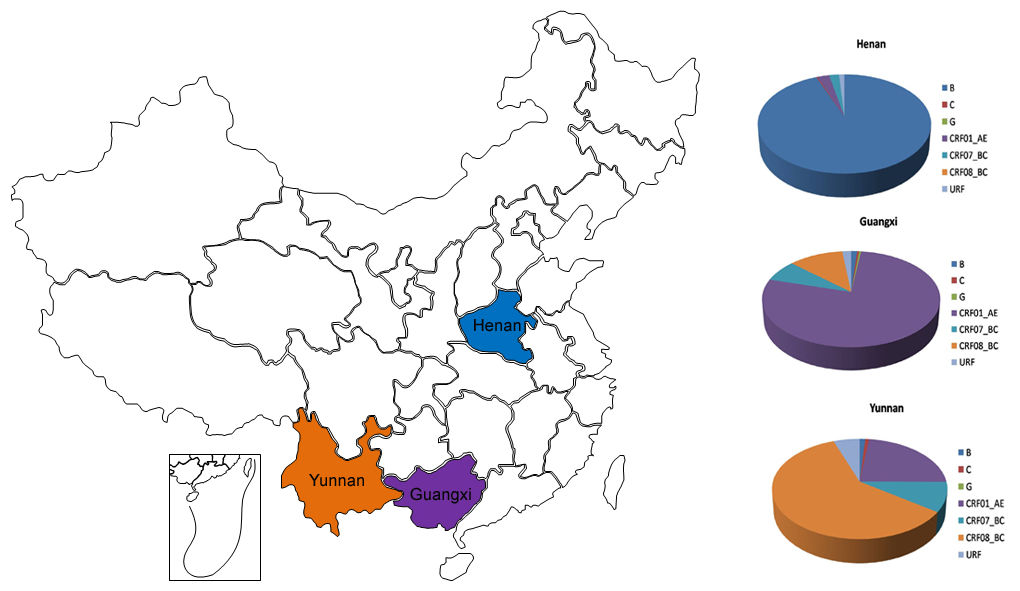
Geographical location of Henan, Guangxi and Yunnan provinces in China and frequency of HIV-1 subtypes. Map of China highlighting Henan, Guangxi and Yunnan provinces were listed in the Left. HIV-1 subtypes were determined by the NCBI viral genotyping tool and phylogenetic analyses, as described in Materials and Methods. The frequencies of HIV subtypes in three provinces were graphied separately in the right column.

From 1159 HIV cases with full length *pol* sequences, 410 (35.4%) subtype B strains, 5 (0.4%) subtype C strains, 2 (0.2%) subtype G strains, 441 (38.1%) CRF01_AE strains, 68 (5.9%) CRF07_BC strains, 205 (17.7%) CRF08_BC strains, and 28 (2.4%) unique recombinant forms (URFs) were identified. Significantly different distributions of HIV main prevalent subtypes, including subtype B, CRF011_AE, CRF07_BC, CRF08_BC and other subtypes (subtype C, G and URF strains were classified into one group to obtain enough samples), in Yunnan, Guangxi and Henan provinces were observed (Table 2). (Pearson chi-square test,χ^2^=1374.240, P=0.000) CRF08_BC was found dominant in Yunnan province which accounted for 59.8% of sequenced isolates from Yunnan. However, CRF01_AE and subtype B dominated separately in Guangxi and Henan provinces, which were responsible for 77.3% and 93.9% of sequenced isolates from those provinces, respectively (Figure 1). The distributions of HIV subtypes in populations infected through different transmitting routes were also compared. CRF01_AE was dominant in HST (Heterosexual transmitted) population, subtype B was dominant in BB (Blood-Born transmitted) population, and CRF08_BC was dominant in IDUs (Intravenous drug users), which suggested the possible association between prevalent subtypes and transmitting routes (Table 2). Multivariate logistic regression was further used to identify significant independent (P<0.05) predictors of different subtypes. To do that, factors including area, sex, ethnic, transmission route and age were included to study the risk may associated with subtypes. The results showed that different subtypes of HIV could be predicted by some factors, including (1) Less subtype B may exist in Guangxi province (OR=0.09, 95% CI: 0.01-0.68, P=0.02) compared with Henan province, but for subtype CRF08_BC, more infections would be observed in Yunnan province (OR=50.46, 95% CI: 4.01-635.4, P=0.00)(2). For subtype CRF07_BC, age may be one risk of infection (OR=1.07, 95% CI: 1.01-1.14, P=0.01).

**Table 2:** Prevalence of HIV subtypes among different populations in Yunnan, Guangxi and Henan.

	HIV-1 subtype							
	B	C	G	CRF01_AE	CRF07_BC	CRF08_BC	URF	Total
Yunnan								
HST	1 (0.4)*	0 (0.0)	0 (0.0)	48 (19.1)	11 (4.4)	99 (39.4)	4 (1.6)	163 (64.9)
MSM	1 (0.4)	0 (0.0)	0 (0.0)	1 (0.4)	0 (0.0)	0 (0.0)	0 (0.0)	2 (0.8)
IDU	1 (0.4)	0 (0.0)	0 (0.0)	1 (0.4)	8 (3.2)	31 (12.4)	4 (1.6)	45 (17.9)
BB	0 (0.0)	0 (0.0)	0 (0.0)	0 (0.0)	0 (0.0)	2 (0.8)	0 (0.0)	2 (0.8)
MTC	0 (0.0)	0 (0.0)	0 (0.0)	2 (0.8)	0 (0.0)	6 (0.4)	0 (0.0)	8 (3.2)
Unknown	0 (0.0)	2 (0.8)	0 (0.0)	6 (2.4)	6 (0.4)	14 (5.6)	3 (1.2)	31 (12.4)
Total	3 (1.2)	2 (0.8)	0 (0.0)	58 (23.1)	24 (9.6)	150 (59.8)	14 (5.6)	251 (100)
Guangxi								
HST	5 (1.0)	2 (0.4)	0 (0.0)	301 (62.6)	25 (5.2)	36 (7.5)	5 (1.0)	374 (77.8)
MSM	0 (0.0)	0 (0.0)	0 (0.0)	6 (1.2)	3 (0.6)	0 (0.0)	1 (0.2)	10 (2.1)
IDU	1 (0.2)	0 (0.0)	1 (0.2)	15 (3.1)	0 (0.0)	12 (2.5)	3 (0.6)	32 (6.7)
BB	0 (0.0)	0 (0.0)	0 (0.0)	0 (0.0)	0 (0.0)	0 (0.0)	0 (0.0)	0 (0.0)
MTC	0 (0.0)	0 (0.0)	0 (0.0)	0 (0.0)	0 (0.0)	0 (0.0)	0 (0.0)	0 (0.0)
Unknown	0 (0.0)	0 (0.0)	1 (0.2)	50 (10.4)	7 (1.5)	7 (1.5)	0 (0.0)	65 (13.5)
Total	6 (1.2)	2 (0.4)	2 (0.4)	372 (77.3)	35 (7.3)	55 (11.4)	9 (1.9)	481 (100)
Henan								
HST	64 (15.0)	1 (0.2)	0 (0.0)	2 (0.5)	3 (0.7)	0 (0.0)	2 (0.5)	72 (16.9)
MSM	9 (2.1)	0 (0.0)	0 (0.0)	4 (0.9)	3 (0.7)	0 (0.0)	0 (0.0)	16 (3.7)
IDU	0 (0.0)	0 (0.0)	0 (0.0)	0 (0.0)	0 (0.0)	0 (0.0)	0 (0.0)	0 (0.0)
BB	303 (71.0)	0 (0.0)	0 (0.0)	3 (0.7)	2 (0.5)	0 (0.0)	2 (0.5)	310 (72.6)
MTC	16 (3.7)	0 (0.0)	0 (0.0)	0 (0.0)	0 (0.0)	0 (0.0)	1 (0.2)	17 (4.0)
Unknown	9 (2.1)	0 (0.0)	0 (0.0)	2 (0.5)	1 (0.2)	0 (0.0)	0 (0.0)	12 (2.8)
Total	401 (93.9)	1 (0.2)	0 (0.0)	11 (2.6)	9 (2.1)	0 (0.0)	5 (1.2)	427 (100)
Total	410 (93.9)	5 (0.4)	2 (0.2)	441 (38.1)	68 (5.9)	205 (17.7)	28 (2.4)	1159 (100)

* Numbers in parentheses indicate the proportion of the HIV-infected in each subgroup as a percentage of the total for the province.

### Prevalence of drug resistant variants

**Figure 2:**
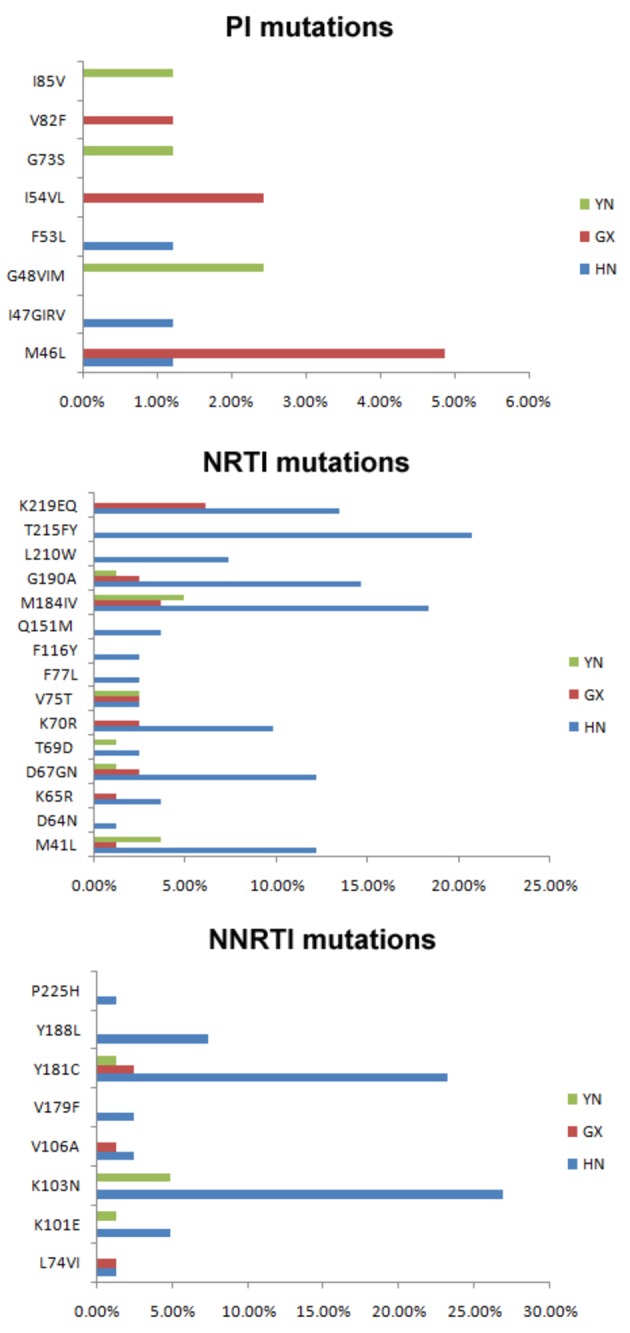
Prevalence of resistant mutations in drug-naïve HIV infected individuals residing in Henan, Guangxi and Yunnan. Mutations presented in strains from Henan, Guangxi and Yunnan were marked separately in blue, red and green.

Drug resistant variants were identified in 82 of the 1159 subjects with full length pol sequences, representing an overall 7.1% (95% CI: 5.7-8.7%) prevalence of drug resistant variants in treatment-naïve individuals. Highest prevalence of HIV drug resistance was found in Henan (12.2%; 95% CI: 9.4-15.7%), followed by Yunnan (5.6%; 95% CI: 3.3-9.2%) and Guangxi (3.3%; 95% CI: 2.0-5.4%). The distribution of drug resistant variants in people infected through different routes was different, with 34 (5.6%) in HST population, 1 (3.6%) in MSMs (Men who have sex with men), 36 (11.5%) in BB individuals, 4 (16%) in HIV-1 infected children who obtained HIV from their mothers. Drug-resistant variants identified in Yunnan and Guangxi were mainly from HST population, while those identified in Henan were mainly from BB population. The proportion of sequences with drug resistant mutations (DRMs) (associated with non-nucleoside reverse transcriptase inhibitors (NNRTIs), nucleoside reverse transcriptase inhibitors (NRTIs) and PIs were 4.9% (57 cases), 4.4% (51 cases) and 1.1% (13 cases) respectively. DRMs associated with resistance to NNRTIs, NRTIs and PIs were also found differently in three provinces. Higher prevalence of NRTIs was identified in Yunnan (9/14, 64.3%) and Guangxi (9/16, 56.3%), while higher prevalence of NNRTIs was identified in Henan (48/54, 88.9%). The pattern of DRMs was also different. 41 patients containing DRMs harbored only one resistant mutation, among of which 12 patients harbored PIs resistant mutations, 12 patient harbored NRTIs resistant mutation, and 17 other patients harbored NNRTIs resistant mutations. All of the other patients displaying DRMs had dual-class mutations to NNRTIs and NRTIs except patient 3668 which contained two PIs resistant mutations. 8 PIs resistant mutations, 15 NRTIs resistant mutations and 8 NNRTIs resistant mutations were identified from 82 patients harboring drug-resistant variants (Figure 2). K103N (31.7%, 26/82) was found most frequently in individuals containing DRMs (Figure 2). Among PIs resistant mutations, M46L, which decreases susceptibility to PIs when present with other mutations, was the most common. The mutation was frequently found in CRF01_AE strains, especially strains prevalent among the MSM population. Further study is needed to illustrate its contribution to resistant phenotype in Chinese CRF01_AE strains.

## Discussion

The prevalence of drug-resistant HIV-1 strains in drug-naïve individuals has important implications for the successful management of ART because it restricts therapy options and increases the risk of treatment failure. In this study, we fulfilled what we believe to be the first province wide surveillance on HIV drug-resistance in treatment-naïve population basing on full length *pol* sequence analysis in three provinces most severely affected by HIV/AIDS in China after the national treatment program. More than 1700 HIV-1 infected cases were enrolled into the study, more than 1100 HIV variants were subjected to drug resistance analysis. The study is likely to be representative of three provinces since samples were selected from all districts with a relative similar ratio. Since participants were not all from new-infected individuals, our data could not represent the transmission of drug-resistant variants. However, the data on prevalence of drug resistant variants in drug-naïve individuals have important implications for clinical therapy.

In this study, CRF01_AE, CRF08_BC and subtype B were found dominant in Guangxi, Yunnan and Henan province respectively, though all subtypes could be identified in each province. Several studies have been fulfilled in the last decade on HIV subtypes epidemic in China [19,20]. The most recent one on a nationwide scale was fulfilled in 2006 [21]. Similar subtype distribution was observed between both studies. However, comparing to the second nationwide molecular epidemiology survey fulfilled in 2001 and 2002 [22], the total ratio of CRF01_AE strain increased from 15.5% to 38.1%. The increase of CRF01_AE strains may be due to the rapid increase of HIV infections transmitted through heterosexual transmission, in which population CRF01_AE was found dominant [13,23-27]. Further studies on pathogenesis of CRF01_AE strains prevalent in China should be fulfilled quickly. In previous studies, two kinds of subtype B strains derived from Thailand B and European or American B strains were demonstrated [21,28]. In our study, the origins of subtype B strains were also analyzed. The result was similar to reports published previously. Only 6 strains among 410 subtype B strains were illustrated to be derived from European and American B strains. Most of them belong to Thai-B original strains which were proved to be prevalent in China.

Highest level of drug resistance was identified in Henan province, where individuals were infected mainly through former unsafe commercial blood and plasma donation (FPD) in the mid-1990s. Antiretroviral drugs were not available in China during the 1990s. So these patients should not be infected with drug resistant viruses at that time. Several reasons may lead to the results. Firstly, the existence of HIV drug resistance in FPDs in this study may due to false inclusion of subjects who had a history of ART. Most farmers who were infected through FPD lack education. They could not distinguish anti-retroviral drugs from other drugs. Re-investigation by phone or interviewing showed that some individuals were unconfirmed with the history of drugs they took. This phenomenon was also observed in a previous study [29]. Secondly, some participants who were infected through intravenous drug use or sexual contact may be reluctant to confess to their mode of transmission due to social stigma. Thirdly, some individuals who acquired HIV through FPD might be super-infected by individuals containing drug-resistant variants [30,31]. Many HIV-positive peoples in Henan married each other after their original spouse died. Anyway, it is ascertained that a high level of drug resistance exist in HIV infected people who reported being drug-naïve in Henan province. So HIV drug resistance of treatment-naïve individuals residing in Henan province should be measured before initiation of ART.

Different DRMs were found prevalent in three provinces, though same combination therapy regimen (AZT+DDI+NVP) was used. NNRTIs resistant mutations were dominant in Henan province, while NRTIs resistant mutations showed higher ratio than NNRTI in Yunnan and Guangxi. The difference was probably due to the significantly different subtype distributions in three provinces. The occurrence of HIV DRMs is subtype specific because of original sequence morphology [32,33]. Furthermore, DRMs may contribute differently to replication fitness of HIV variants belonging to different linkage. In a previous study, we found that Y181C (NNRTIs resistant mutation) could significantly increase the replication capacity of subtype B strains [34]. That may be the reason why NNRTIs resistant mutations were more common than NRTIs resistant mutations in Henan province. However, the effect of this mutation on other subtype variants was unknown. Further studies are needed to investigate the effect of DRMs on the replication fitness of HIV strains belonging to different subtypes.

In this survey, unequal distributions of HIV subtypes and drug-resistance were found in treatment-naïve individuals among three provinces with the most severe HIV epidemic in China. Although the rate of DRMs in three provinces as a total was relatively low compared to those in developed countries [35,36], a high prevalence of drug-resistant variants was found in some specific areas, for example in Henan province. Resources for antiretroviral drugs are limited in China. There is a concern that the prevalence of antiretroviral drug resistance will compromise the effect of current regimens and give rise to treatment failure. The results make urgent the testing of HIV drug-resistance in drug-naïve people before the start of free HAART in China. The identification of PI resistant strains in all three areas implicated that the free second line drugs in which a PI drug was included should be evaluated before the comprehensive application. In the absence of universal baseline resistance testing, more studies should be performed to provide different regimens according to distinguishing epidemic status in different areas in China.
